# Accessibility of Digital Financial Applications for People With Visual Impairment: Scoping Review

**DOI:** 10.2196/82315

**Published:** 2026-06-23

**Authors:** Louise Puli, Lars Kooijman, Tanjila Kanij, Charmine Hartel, Abu Zafar M Shahriar, Kristian Rotaru

**Affiliations:** 1 Department of Banking and Finance, Monash Business School, Monash University, 900 Dandenong Road, Caufield East, Victoria, 3145, Australia, 61 099032211; 2 Rehabilitation, Ageing and Independent Living Research Centre, School of Primary and Allied Health Care, Monash University, Frankston, Victoria, Australia; 3 Opportunity Tech Lab, Monash Business School, Monash University, Caufield East, Victoria, Australia; 4 Office of the Chief Forensic Scientist, Victoria Police Forensic Services Department, Macleod, Victoria, Australia; 5 Department of Computing Technologies, School of Science, Computing and Emerging Technologies, Swinburne University of Technology, Hawthorn, Victoria, Australia; 6 Department of Management, Monash Business School, Monash University, Caufield East, Victoria, Australia; 7 Department of Accounting, Monash Business School, Monash University, Caufield East, Victoria, Australia; 8 School of Psychological Sciences, Monash University, Clayton, Victoria, Australia

**Keywords:** visual impairment, accessibility, financial technology, user-centered design, assistive technology, digital inclusion, PRISMA, consumer empowerment, Preferred Reporting Items for Systematic Reviews and Meta-Analyses

## Abstract

**Background:**

Routine financial activities are now conducted primarily through digital channels. Many such systems remain inaccessible to more than 2.2 billion people globally living with vision impairment, limiting independent financial management. Constrained access can create financial strain and social disadvantage, reducing access to health-enabling resources, and contributing to avoidable health inequities.

**Objective:**

This scoping review maps evidence on the accessibility of digital financial services for individuals with visual impairment (VI) as a digital determinant of health. We synthesized barriers and facilitators, characterized study designs, settings, and populations, and identified evidence gaps to inform inclusive design, digital health research priorities, and policy.

**Methods:**

A scoping review was conducted using the Joanna Briggs Institute framework and reported in line with PRISMA-ScR (Preferred Reporting Items for Systematic Reviews and Meta-Analyses extension for Scoping Reviews) guidelines. Eight databases (PubMed, MEDLINE, CINAHL, Scopus, Web of Science, Business Source Complete, ProQuest, and IEEE Xplore) were searched for peer-reviewed papers in English published between 1995 and 2026. Searches featured controlled vocabulary and free-text terms structured in 3 conceptual blocks (VI, digital financial services, and accessibility or usability). A random sample of 20% of titles, abstracts, full texts, and included studies was independently screened or charted by 2 reviewers to calibrate decisions; the remainder were screened and charted by a single reviewer. Data were charted using a standardized extraction form, and results were synthesized descriptively and thematically.

**Results:**

Twenty-three studies met the inclusion criteria. Studies were conducted across 12 countries, with the largest number from India (n=7), Indonesia (n=2), Thailand (n=2), and the United States (n=2). Study designs included qualitative studies (n=6), mixed methods studies (n=1), cross-sectional studies (n=4), nonrandomized experimental studies (n=2), and technical or design-focused evaluations (n=6). One study was a large population survey (n=19,136), and the remaining studies with human participants had sample sizes ranging from 4 to 36 participants. Accessibility barriers were reported across all platform types, with authentication-related barriers described in 18 studies and screen reader incompatibility in 17 studies. Reported barriers included reliance on sighted assistance for tasks such as login, verification, and payments, compromising privacy and independence. Facilitators included assistive technology support, logical navigation order, nonvisual feedback mechanisms, and accessible authentication alternatives. Evidence mapping revealed recurrent barrier patterns across Android, iOS, and web platforms. No longitudinal or intervention-based evaluations were identified.

**Conclusions:**

This review provides a focused synthesis of accessibility evidence at the intersection of digital financial services and VI, a domain addressed by neither prior digital accessibility reviews nor financial inclusion for people with disabilities. Authentication methods, interface labeling, and navigation were identified as persistent cross-platform accessibility barriers. The findings carry implications for financial technology developers, accessibility auditors, and policymakers implementing accessibility legislation and extend the digital determinants of health framework by demonstrating how inaccessible financial technology may compound health inequities.

## Introduction

Globally, 2.2 billion people live with some form of visual impairment (VI), including blindness or low vision [1]. Individuals with VI consistently report reduced quality of life and social exclusion, impacting access to education, employment, housing, and essential services [1-3]. As digital banking, particularly mobile- and web-based apps, becomes the predominant means of accessing financial services [4], concern is increasing that people with VI face substantial barriers to digital financial inclusion [3].

Access to financial resources is recognized as a social determinant of health [5]. Accordingly, access to financial services mediated through digital technologies can affect health outcomes [6]. These interactions, described as digital determinants of health [6], warrant close examination to identify how digital systems create or reinforce barriers for people with disabilities. Consistent with this perspective, Richardson et al [7] proposed a comprehensive digital health equity framework that identifies access to digital tools, including financial management platforms, as a determinant operating across individual, interpersonal, community, and societal levels. Empirical evidence supports this framing. Naveenan et al [8] showed that digital inclusion moderates the relationship between financial inclusion and health outcomes in developing countries, suggesting that inaccessible digital financial services may attenuate the health benefits of financial participation.

Barriers for people with VI commonly arise from a misalignment between user needs and visually dominant design practices in financial technologies, which are typically developed for visual rather than multisensory interaction [9]. VI spans a wide spectrum, from low vision to complete blindness, necessitating tailored design approaches. For instance, individuals with low vision often rely on adjustable font sizes, high-contrast displays, and customizable layouts to use digital banking apps effectively [3]. In contrast, people who are blind typically navigate digital environments using assistive technologies such as screen readers, voice commands, tactile input devices, or refreshable Braille displays [10]. However, despite the existence of assistive technologies, these tools are frequently unavailable, particularly in low- and middle-income settings, and are often difficult to use in practice [11]. Even when assistive technologies are accessible, many digital financial apps remain poorly designed, with limited compatibility that renders essential financial tasks difficult or impossible to complete independently [1-3,9,11].

Beyond interface-level barriers, engagement with digital financial apps is shaped by user-specific factors such as digital and financial literacy, familiarity with assistive technologies, and privacy concerns [11]. Security mechanisms that rely primarily on visual input, including CAPTCHA, biometric prompts, and multifactor authentication, often lack accessible alternatives. Although intended to enhance security, such measures can inadvertently exclude people with VI and reinforce digital health inequities [3]. In this context, access to financial services represents not only a technical issue but also a matter of human rights and social inclusion [1]. Financial inclusion supports at least 5 United Nations Sustainable Development Goals, including poverty reduction, gender equality, and reduced inequalities [12]. Inaccessible digital platforms risk exacerbating existing disparities and undermining broader development objectives [11].

Inclusive, user-centered design and participatory co-design with people with VI are widely recognized as essential for addressing accessibility barriers in digital platforms [13,14]. Kim [14] demonstrated that designers’ mental models often differ fundamentally from those of users with visual disabilities, a disconnect that may help explain persistent accessibility failures in financial apps. Research-informed personas and co-design approaches have been used to guide the development of more inclusive systems, emphasizing simplified navigation, nonvisual authentication options, and consistent assistive technology compatibility [15,16]. To the best of our knowledge, no review has focused specifically on the accessibility of digital financial services for people with VI. Although Puli et al [12] examined financial inclusion for disability more broadly, their review did not address VI-specific barriers.

Several reviews have examined the broader intersection of information and communication technology (ICT) and VI [17-21]. Ashraf et al [17], for example, conducted a systematic review of ICT apps designed to support people with VI across multiple domains, reporting positive effects on self-esteem but without quantifying broader life impacts or addressing financial inclusion specifically. Similarly, Al-Razgan et al [20] reviewed usability barriers in mobile apps for people with VI across themes such as assistive devices, screen layout, and audio guidance, but did not examine financial technologies in detail. Hamideh Kerdar et al [21] identified systemic accessibility barriers in digital apps for people with VI, without focusing on financial services. Jonsson et al [22] found limited consideration of sensory impairments in digital health service design, while Fayyad and Al-Sinnawi [23] reported that banks in Palestine remained reluctant to implement accessible services for people with VI despite policy commitments.

Collectively, this literature indicates that although digital accessibility and financial inclusion have been studied extensively in isolation from each other, their intersection remains underexplored. Existing research has largely examined accessibility challenges in general digital environments or financial inclusion at a population level without identifying the specific mechanisms through which digital financial systems exclude people with VI. As a result, there is limited evidence on the barriers and facilitators shaping access to digital financial tools, particularly across different platforms, user contexts, and stages of the transaction process. This gap is significant because digital financial tools have the potential to enhance autonomy and financial participation, yet inaccessible systems may instead reinforce dependence, limit privacy, and exacerbate existing social and health inequities [12].

To address this gap, this scoping review aims to (1) map the characteristics, settings, and populations of existing research; (2) synthesize reported barriers and facilitators affecting accessibility of digital financial services; (3) examine methodological approaches in this field; and (4) identify evidence gaps to inform future research and policy. The findings are intended to support more inclusive digital financial and health-enabling technologies.

## Methods

### Study Design

This scoping review follows the methodological framework outlined by the Joanna Briggs Institute for scoping reviews and is reported in accordance with the PRISMA-ScR (Preferred Reporting Items for Systematic Reviews and Meta-Analyses extension for Scoping Reviews) checklist (Checklist 1)[24]. The PRISMA-ScR checklist was verified against the paper on revision, and all items are addressed within the Methods and Results sections.

### Protocol and Registration

A review protocol was developed a priori by the review team to define the review questions, eligibility criteria, search strategy, and data charting framework. No substantive deviations from the protocol occurred following commencement of the review. The review protocol was not registered prospectively.

### Research Questions

This scoping review was designed to systematically map the literature on the accessibility of digital financial services for people with VI. The review addressed the following questions:

1. What types of evidence exist regarding the accessibility of digital financial services for people with VI?
2. What are the primary barriers and facilitators identified in the literature?
3. What are the characteristics of research conducted in this field (eg, study designs, settings, and populations)?
4. What gaps exist in current evidence to guide future research and policy?

### Eligibility Criteria

Eligible studies included (1) studies reporting end user data from people with VI and (2) technical or design-focused evaluations directly relevant to the accessibility or usability of digital financial apps (eg, assistive technology compatibility, text scaling, or authentication workflows). Eligible study designs included qualitative studies, cross-sectional studies, experimental or quasi-experimental studies, and technical or prototype development studies relevant to the accessibility of digital financial apps. We included technical evaluations because accessibility barriers in financial apps can arise from implementation choices (eg, text scaling, labeling, and authentication workflows) even when end user testing is absent.

The search encompassed publications from 1995 through February 2026. This extended timeframe was chosen to capture early research on accessibility in digital finance and related sociotechnical systems, such as web-based banking and authentication, that preceded contemporary mobile banking. In practice, included studies clustered from 2011 onward, corresponding with the rise of modern mobile financial apps.

Studies were excluded if they did not focus on VI or digital financial services, lacked an accessibility or usability component, or were not peer-reviewed. Non-English publications were excluded because of the language limitations of the review team. Publications without sufficient methodological detail to allow extraction of study characteristics or accessibility findings (eg, abstracts without full text) were also excluded.

### Information Sources

Searches were initially conducted in June 2024 and were rerun on February 27, 2026, to capture newly published studies before final screening. The following databases were searched: PubMed, MEDLINE, CINAHL (EBSCOhost), Scopus, Web of Science, Business Source Complete (EBSCOhost), ProQuest, and IEEE Xplore. No additional information sources (eg, gray literature, hand-searching, or contact with authors) were used.

### Search Strategy

The search strategy combined controlled vocabulary (eg, MeSH [Medical Subject Headings] in MEDLINE and CINAHL headings, where appropriate) and free-text terms across 3 conceptual blocks, combined using Boolean operators. The three blocks were (1) VI or blindness; (2) accessibility, usability, assistive technology, and common barriers (including authentication-specific terms such as CAPTCHA, one-time password [OTP], and biometric); and (3) digital financial services and applications (including mobile banking, fintech, digital wallets, and mobile payments).

Within each conceptual block, synonyms and related terms were combined using OR, and the 3 blocks were combined using AND. Truncation and phrase searching were applied where supported by individual databases.

The original strategy used 4 conceptual blocks; this was revised to 3 in line with the Cochrane Handbook guidance (Section 4.4.2) to improve sensitivity by consolidating related concepts and reducing overly restrictive AND combinations. The revised search was rerun across all databases on February 27, 2026.

The complete search strategies for all databases, including search terms and Boolean operators, are provided in Multimedia Appendix 1. No geographic limits were applied. We limited our search to English-language peer-reviewed publications because the review team lacked shared fluency in additional languages, and the aim was to map published research with reproducible methods and accessible full texts within the review timeframe.

### Search Reporting

Search methods were additionally reported in accordance with the PRISMA-S [25] extension for literature search reporting. Full search strategies for all databases, including exact search strings, Boolean operators, limits, and database-specific adaptations, are provided in Multimedia Appendix 1.

Searches were limited to English-language, peer-reviewed publications published between 1995 and 2026. Records were imported into Covidence (Veritas Health Innovation Ltd), and duplicates were removed before screening.

No additional search methods (eg, citation searching, hand-searching of journals, or gray literature searches) were undertaken.

### Selection of Sources of Evidence

Search results were imported into Covidence for deduplication and screening. To ensure consistent application of eligibility criteria, a random 20% sample of titles and abstracts was independently screened by 2 reviewers. Discrepancies were discussed, and the screening approach was calibrated before screening the remaining records, which were assessed by a single reviewer.

Full-text papers deemed potentially eligible were retrieved and evaluated against the inclusion criteria. As in the title and abstract screening stage, 20% of full-text papers were independently assessed by 2 reviewers to verify consistency, with the remainder reviewed by a single reviewer. Disagreements during dual screening were resolved through discussion, with consultation from a third reviewer when consensus could not be reached.

Full search strategies for each database, including exact search strings, limits, and dates searched, are provided in Multimedia Appendix 1.

### Data Charting Process

A standardized data charting form was developed through team consultation and piloted on 2 included studies before full data extraction. To ensure consistency, 20% of studies were independently charted by 2 reviewers, with the remaining studies charted by a single reviewer using the finalized framework. Any discrepancies identified during dual charting were resolved through discussion and consensus.

### Data Items

Data items were defined a priori and extracted using a standardized charting form. Extracted variables included bibliographic details (author, year, and country); study characteristics (study design); population details (type and severity of VI and demographics); type of financial application studied; accessibility features examined; reported user needs, barriers, and outcomes; and evaluation focus (end user study, technical evaluation, or mixed).

These variables were selected to support descriptive mapping of accessibility barriers and facilitators across platforms and study contexts.

### Critical Appraisal of Individual Sources of Evidence

Consistent with scoping review methodology, formal critical appraisal of individual sources of evidence was not undertaken [24]. The aim of this review was to map the extent, characteristics, and nature of the available evidence rather than assess the methodological quality or risk of bias of the included studies.

### Data Synthesis

Charted data were synthesized descriptively by one author (LP) and discussed with the full research team to inform interpretation. Study characteristics were summarized using counts and frequencies. Accessibility barriers and facilitators were grouped using inductive thematic categorization based on reported findings, without formal qualitative coding. Evidence mapping was used to visualize the distribution of barrier types across platform contexts.

### Ethical Considerations

Ethics approval was not required for this study because it involved the analysis and synthesis of data from publicly available published literature and did not involve the collection of primary data from human participants.

## Results

### Selection of Sources of Evidence

The final search, conducted on February 27, 2026, identified 1719 records, including 1134 from Scopus, 484 from PubMed, 75 from ProQuest, 10 from CINAHL, 9 from Business Source Complete, 6 from Web of Science, and 1 from IEEE Xplore. There were 218 duplicates, leaving 1501 records for title and abstract screening.

**Figure 1.**
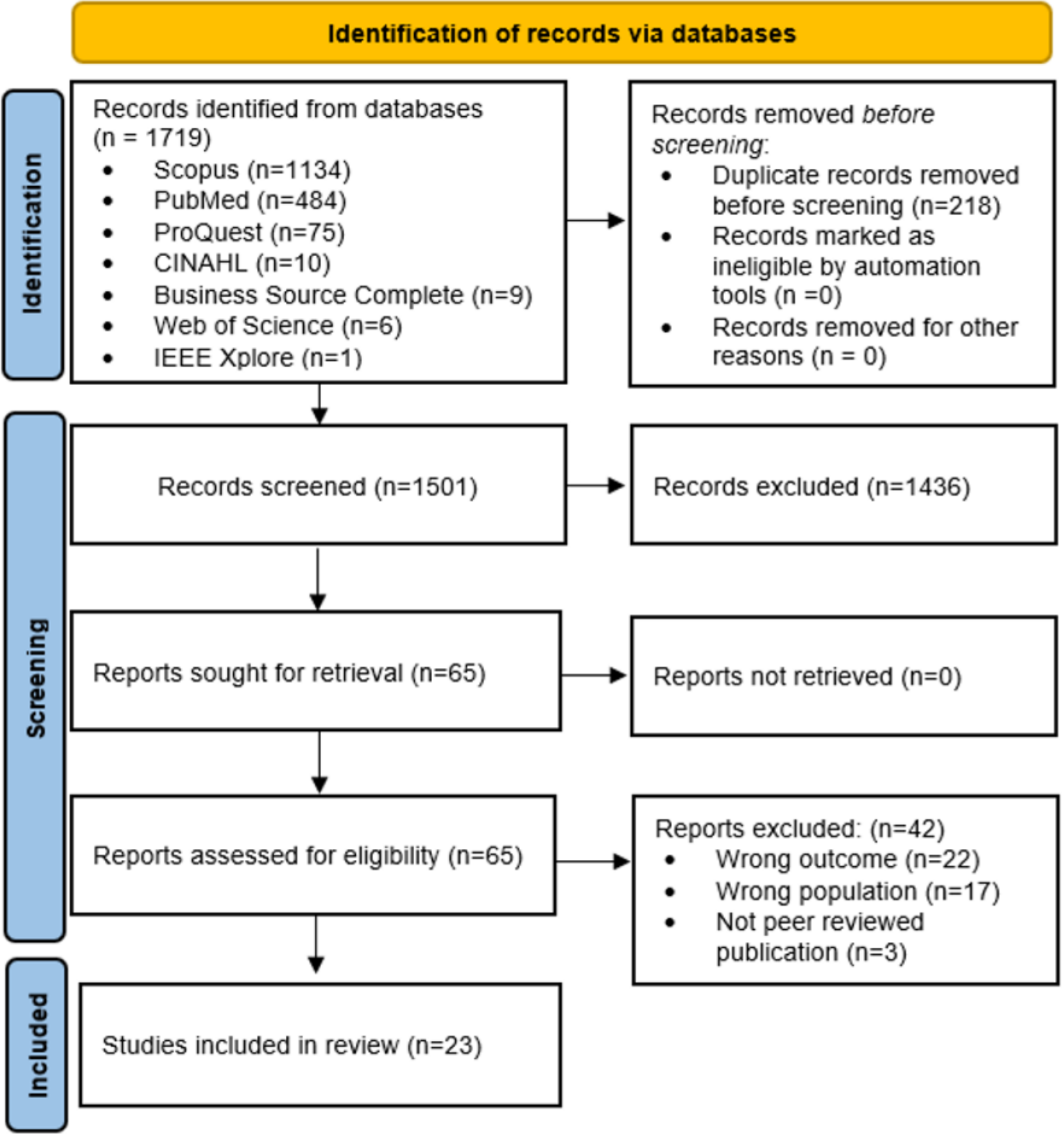
PRISMA-ScR (Preferred Reporting Items for Systematic Reviews and Meta-Analyses extension for Scoping Reviews) flow diagram summarizing the scoping review search results.

During title and abstract screening, 1436 records were excluded, and 65 full-text papers were sought and assessed for eligibility; all were retrieved. Of these, 42 full-text papers were excluded. A total of 23 studies were included in the review. There were no ongoing studies and no studies awaiting classification. The study selection process is summarized in the PRISMA-ScR flow diagram (Figure 1).

### Characteristics of Sources of Evidence

**Figure 2.**
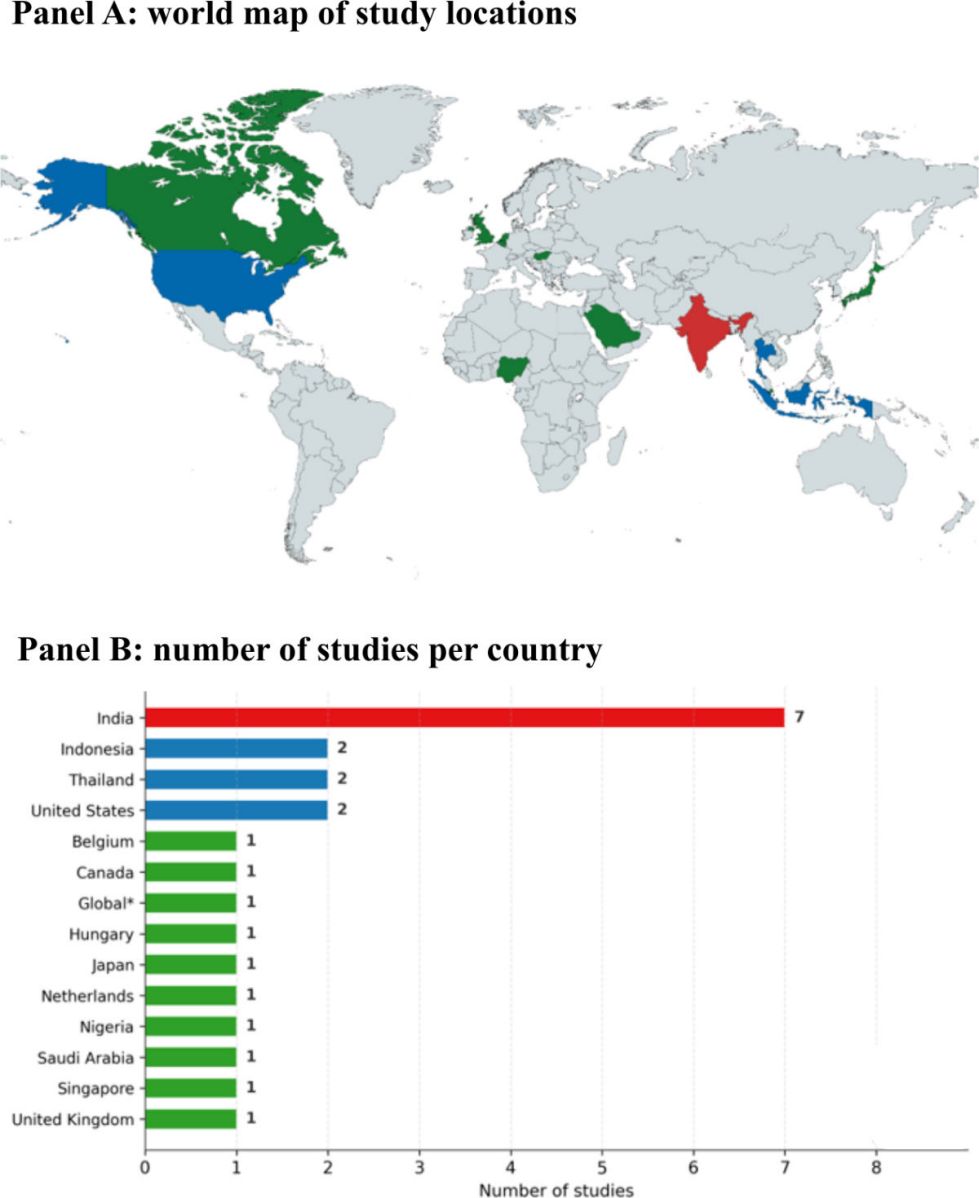
Geographic distribution of the 23 included studies. (A) World map showing the countries in which the included studies were conducted. Colors indicate study count per country: red (n=7), blue (n=2), and green (n=1). Custom map image created with Mapchart [45]. (B) Corresponding bar chart of study counts by country. India accounted for nearly one-third of all included studies (n=7), followed by Indonesia, Thailand, and the United States (n=2 each). The remaining 9 countries contributed 1 study each. One additional study (Alshayban and Malek [15]) was global in scope and is not mapped to a specific country.

Table 1 provides the characteristics of the included studies [9,10,13,15,26-44]. The 23 included studies were published between 2011 and 2026. Figure 2A provides the geographic distribution of the included studies. As shown in Figure 2B, the largest number of studies was conducted in India (n=7). Studies were also conducted in the United States (n=2), Thailand (n=2), Indonesia (n=2), Japan (n=1), Belgium (n=1), Nigeria (n=1), the Netherlands (n=1), Saudi Arabia (n=1), the United Kingdom (n=1), Singapore (n=1), Canada (n=1), and Hungary (n=1), alongside 1 study described as global in scope.

Study designs included qualitative studies (n=6), mixed methods study (n=1), cross-sectional studies (n=4), nonrandomized experimental studies (n=2), design and prototype evaluations (n=4), and technical evaluations without human participants (n=6).

**Table 1.** Characteristics of the 23 included studies on the accessibility of digital financial apps for people with visual impairment, published from 2011 to 2026. Studies were identified through a systematic search of 8 databases. For each study, the table provides the lead author and year, country, and setting; study design; participant number; characteristics of participants (or data sources for technical studies), type of visual impairments addressed, description of the financial application or system evaluated, and assistive technology used or assessed.

Study and year	Country	Study design	Participant number	Visual impairment (as described by the study)	Financial application description	Assistive technology use
Afandi et al (2026) [26]	Indonesia	Qualitative	4	Blind and partially sighted	Inability to use mobile banking apps due to a lack of screen reader compatibility	Limited screen reader use
Alayed (2025) [27]	Saudi Arabia	Mixed methods	12	Blind	SNB^a^, AlRajhi, and Riyad Bank mobile banking apps were evaluated for accessibility	Participants used VoiceOver (iOS) or TalkBack (Android) screen readers
Alnfiai and Sampalli (2019) [10]	Canada	Nonrandomized experimental	10	Blind or visually impaired	BraillePassword: an accessible web authentication technique on touchscreen devices (mobile, tablet, or computer)	Participants used screen readers and headphones to prevent aural eavesdropping
Alshayban and Malek (2022) [15]	Global	Empirical study	No end user participants	Low vision	Automated software tool AccessiText, developed by Alshayban and Malek [15], for detecting text accessibility issues in Android apps arising from incompatibility with the Text Scaling Assistive Service	Android text scaling accessibility (data analysis), iOS Dynamic Type referenced where applicable, AccessiText tool developed for Android only
Angsupanich and Matayong (2024) [28]	Thailand	Prototype development	5	Visual impairments	A mobile app for currency recognition	Google text-to-speech
Braeken (2017) [29]	Belgium	Cryptographic system design and formal security evaluation	No end user participants	Visual impairments	Smartphone used as a payment proxy device	Not reported
Branham and Kane (2015) [30]	United States	Qualitative	40 (20 sighted and 20 blind participants)	Blind and sighted pairs	Collaborative accessibility using a sighted peer support	All participants with visual impairment used screen readers
Christy and Pillai (2021) [31]	India	Cross-sectional	15	Blind and low vision	The study included 57 apps across various categories, such as online shopping and transport	Assistive gestures used in iOS and Android; screen readers (TalkBack and KNFB Reader), voice assistants (Siri and Google Assistant), and magnifiers
Fenwick et al (2022) [32]	Singapore	Cross-sectional	27	Age-related macular degeneration, including polypoidal choroidal vasculopathy.	Identifying impact of age-related macular degeneration on age-related quality of life, including financial well-being, using computerized adaptive testing	Not reported
Gopalakrishnan et al (2022) [33]	India	Pilot study with focus groups	15	Peripheral and central field loss	A virtual reality bank to assess the functional performance of people with low vision compared with those with normal vision	Virtual reality headset to access virtual bank
Kameswaran et al (2018) [34]	India	Qualitative	32	Blind and low vision	Digital ride-hailing services	All participants used smartphones; 25 used Android phones with TalkBack screen readers, and 7 iPhones with VoiceOver and Google Maps
Kameswaran and Hulikal Muralidhar (2019) [35]	India	Qualitative	30	Visual impairments	Digital payment interface while accessing payment on rideshare apps in India	Twenty-five participants used screen readers built into their Android phone (TalkBack or VoiceOver)
Kameswaran et al (2023) [36]	India	Qualitative	30	Blind and low vision	Internet banking, mobile banking, and third-party banking. Among participants, 25 used internet banking and mobile banking, 27 used digital payments, and 3 did not use digital banking because they were uncomfortable with technology	All participants used screen readers for computers and mobile phones. Among participants, 20 used Android mobile phones (with TalkBack), 7 used iPhones (with VoiceOver), and 3 used both an iPhone and an Android phone
Maesaka (2015) [37]	Japan	Design case study	5	Color vision weakness (color vision deficiency), age-related vision deterioration, glaucoma, cataract, and general accessibility considerations.	Smartphone-oriented internet banking system designed by NEC^b^ compliant with JIS X 8341-3 classes A and AA	Voice reading software
Okonji et al (2020) [38]	Nigeria	Cross-sectional	98	Visually impaired with best corrected visual acuity lower than 6/36 (20/120), indicating moderate-to-severe vision loss.	Online banking, shopping, and bill payment	Screen readers such as Dolphin-Supernova (Dolphin Computer Access Ltd), ZoomText (Freedom Scientific), and JAWS (Freedom Scientific)
Radványi et al (2011) [39]	Hungary	Nonrandomized experimental	5	Visually impaired	A mobile app for banknote recognition	Bionic Eyeglass prototype
Ramani et al (2023) [40]	India	Descriptive design and prototype development	No end user participants	Blind	Mobile app prototype developed to recognize Indian currency in real time and provide audio output of the detected note	Smartphone or tablet with camera
Reyes-Cruz et al (2022) [13]	United Kingdom	Ethnomethodological study	10	Blind and partially sighted	Analyzed video-recorded demonstrations involving participants with visual impairments, HCI^c^ to identify accessibility issues	VoiceOver (Apple), TalkBack (Google), JAWS (Freedom Scientific), Siri (Apple), Seeing AI (Microsoft), KNFB Reader (Sensotec NV), text detection, facial recognition, and light detection
Singh et al (2024) [9]	India	Qualitative	15	Visually disabled	Application to support digital payments on Google Pay, PhonePe, and Paytm (One97 Communications)	All participants used smartphones with screen reader assistive tools
Sofiyati et al (2024) [41]	Indonesia	Prototype development	No end user participants	Low vision	CurrencyNet is a Rupiah banknote recognition application	LoVi^d^ application provides voice feedback and uses a smartphone camera; designed for use on common smartphones
van der Cruijsen and Reijerink (2024) [42]	Netherlands	Cross-sectional	19,136 respondents	Blind or visually impaired (2.1% of respondents), deaf or hearing impaired, limited or no hand function, mild intellectual disability, and mobility impairments (walking difficulty or wheelchair).	Description of payment environment: high digitalization (contactless cards and smartphones); 20% of POS^e^ transactions paid with cash (2022); near-universal card and internet access; high acceptance of cash (96%) and debit cards (92%). Dependent variables (cash importance measures): share of POS transactions paid with cash, uses cash only, cash-dependent (“cannot do without cash”), and cash preference	Not reported
Yang et al (2014) [43]	United States	Prototype development	No end user participants	General visually impaired	A Money Reader Android app for recognizing US dollar banknotes, custom-built by Yang et al [43]	Android accessibility features (TalkBack and Explore by Touch) and a text-to-speech engine for audio output
Yawai et al (2024) [44]	Thailand	Prototype development	No end user participants	General visually impaired	A Thai banknote and coin recognition application	Real-time voice output and audio feedback

a SNB: Saudi National Bank.

b NEC: Nippon Electric Company.

c HCI: human-computer interaction.

d LoVi: low vision.

e POS: points of sale.

### Participant Characteristics and Data Sources

**Figure 3.**
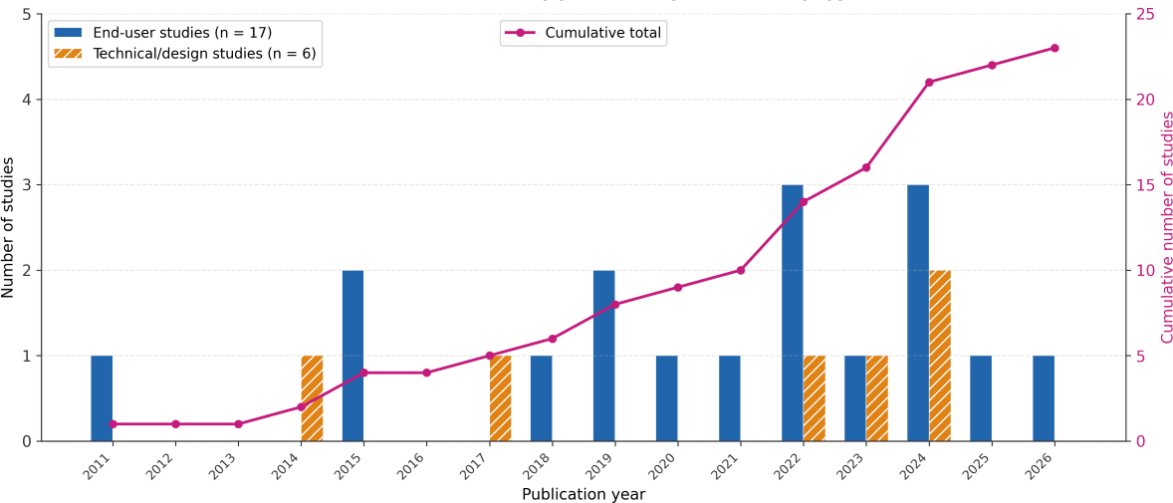
Distribution of the included studies (n=23) by publication year and study type. Studies are grouped by type: end user studies involving human participants (n=17; solid blue bars) and technical or design-focused evaluations without human participants (n=6; hatched orange bars). The cumulative total is shown as a line (pink). Publication activity was sparse from 2011 to 2020, with no more than 2 studies per year. A marked increase occurred from 2022 onward, with more than 73.9% (17/23) of the included studies published between 2022 and 2026.

Of the 23 studies, 17 included human participants, and 6 were technical evaluations without human participants. Among the 17 studies with human participants, 14 focused directly on people with VI and 3 enrolled broader samples, such as blind and sighted pairs, respondents with VI as a subgroup, or related clinical populations. As shown in Figure 3, publication activity in this field was sparse until 2022, after which a marked increase occurred, with more than 70% of the included studies published from 2022 onward.

Excluding 1 large population survey (van der Cruijsen and Reijerink [42], n=19,136 and 2.1% with VI), the remaining studies with human participants had sample sizes ranging from 4 to 98. The population survey is reported separately from the smaller end user studies to avoid distorting the participant summary. Low-vision participants were included in 6 of 17 studies, 3 enrolled participants who were blind and participants with low vision, and 4 used nonspecific descriptors (eg, “visually impaired”) without severity detail.

Specific diagnoses were inconsistently reported. Where described, conditions included age-related macular degeneration, glaucoma, cataracts, retinitis pigmentosa, peripheral field loss, and central field loss.

### Synthesis of Results

#### Overview

Of the 23 included studies [9,10,13,15,26-44], 6 studies used qualitative research designs, including semistructured interviews, focus groups, and related observational approaches. One study (Afandi et al [26]) was classified as qualitative based on its interview-based methodology, although the original extraction described it as a case-control study. One study used a mixed methods design combining qualitative approaches with observational analyses. Four studies used cross-sectional designs, including a population survey on digital payment behavior and surveys of technology use among people with VI. Two studies used nonrandomized experimental designs to evaluate accessible authentication methods and tactile currency recognition tools. Four studies used design or prototype evaluation approaches involving human participants, including prototype development with end user testing, virtual reality pilot testing, a design case study, and an ethnomethodological analysis of assistive technology use. The remaining 6 studies were technical evaluations without human participants, including prototype development for currency recognition and accessible payments (n=4), automated accessibility evaluation (n=1), and cryptographic system design (n=1).

#### Assistive Technology Use

Assistive technology use was reported in 14 of the 17 end user studies. Screen readers were the most frequently described assistive technologies, with TalkBack (Android; Google) explicitly reported in 6 studies and VoiceOver (iOS; Apple) reported in 5 studies; 4 studies reported the use of both platforms. In addition to screen readers, several studies described the use of other assistive technologies, including optical character recognition readers, seeing artificial intelligence, KNFB Reader, refreshable Braille displays, screen magnification tools, and voice assistants.

The 6 technical evaluations without human participants assessed a variety of tools, such as authentication techniques, automated accessibility detection tools, currency recognition algorithms, prototype apps, and payment system architectures. These studies often evaluated how these technologies interacted with assistive technology. Furthermore, these studies predominantly examined system design or functionality relevant to accessibility but did not always involve direct participation of users with VI.

#### Accessibility and Usability Barriers

Barriers related to screen reader compatibility and interface labeling were reported in 17 of 23 studies [9,10,13,15,26-44]. These included missing or incorrect screen reader labels, unlabeled buttons or form fields, and unclear prompts. Issues related to navigation flow and focus order were described in 15 studies, including difficulties moving sequentially through screens or completing multistep workflows. Complex interface layouts that limited efficient nonvisual navigation were reported in 12 studies.

Authentication-related barriers were reported in 18 studies. Visual CAPTCHA challenges were described in 14 studies, and OTP workflows requiring app switching were reported in 13 studies. Biometric authentication mechanisms that were not consistently accessible were described in 9 studies. Additional authentication-related issues included session timeouts in 6 studies, personal identification number entry difficulties in 7 studies, and inconsistent or inaccessible error messaging in 8 studies.

Other usability barriers described across studies included missing alternative text for images in 10 studies, layout distortions when text scaling was applied in 7 studies, auto-scrolling content or advertisements in 5 studies, limited audio or haptic feedback in 6 studies, and technical failures such as application crashes or freezing in 4 studies.

#### Accessibility Features and Facilitators

Accessibility features were reported in 19 [9,10,13,15,26-28,30,31,34-38,41-45] of 23 included studies [9,10,13,15,26-44]. Table 2 organizes the reported barriers, enablers, and evidence gaps across 5 stages of the user journey (ie, authentication, navigation, transaction, confirmation, and ongoing use), showing where the evidence is concentrated and where gaps remain. Among the most frequently reported features, screen reader compatibility was described in 15 studies, while provision of alternative text for images or interface elements was reported in 9 studies. Keyboard or gesture-based navigation options were described in 7 studies, and voice-based interaction features were reported in 6 studies.

Haptic feedback for confirmation of transactions or authentication steps was described in 5 studies. Alternative authentication mechanisms designed to reduce reliance on visual input (eg, tactile or nonvisual authentication approaches) were evaluated in 4 studies.

**Table 2.** Accessibility barriers, enablers, and evidence gap matrix for digital financial services across the user journey. The matrix maps accessibility barriers, enablers, and evidence gaps identified across the 23 included studies along 5 stages of the digital financial services user journey: authentication, navigation, transaction, confirmation, and ongoing use. For each stage, the specific barriers reported in the literature, the enablers or design strategies proposed or evaluated, and the gaps where empirical evidence remains limited or absent are presented. The framework illustrates how barriers compound across the user journey rather than occurring in isolation, and highlights the stages where future research is most needed.

Factor	Authentication	Navigation	Transaction	Confirmation	Ongoing use
Barriers	CAPTCHA^a^, OTP^b^ requiring app switching, and inaccessible biometric authentication.	Poor focus order, complex layouts, multistep workflows, and screen reader incompatibility.	Unlabeled fields, inaccessible forms, and missing alternative text.	Lack of audio or haptic feedback and unclear error messages.	Updates are breaking accessibility, and app crashes or freezing.
Enablers	Gesture-based authentication and alternative CAPTCHA.	Logical navigation structure and screen reader optimization.	Proper labeling and accessible form design.	Audio feedback, haptic confirmation, and clear error messaging.	Ongoing accessibility testing and compatibility monitoring.
Evidence gaps	Lack of evaluation of accessible authentication methods in real-world financial apps; limited testing of alternatives to CAPTCHA, OTP, and biometrics; and absence of security–accessibility trade-off analysis.	Limited empirical evaluation of navigation improvements (eg, focus order and layout simplification) on task completion and efficiency, and lack of standardized accessibility benchmarks across platforms.	Minimal evidence on end-to-end transaction completion success, limited testing of accessible form design in real financial workflows, and absence of error recovery and usability studies.	Lack of research on feedback mechanisms (audio and haptic) and their impact on user confidence, error detection, and transaction safety.	No longitudinal studies examining how app updates affect accessibility over time, and the absence of evidence on regression testing practices or long-term compatibility monitoring with assistive technologies.

a CAPTCHA: Completely Automated Public Turing test to tell Computers and Humans Apart.

b OTP: one-time password.

#### Reported Recommendations and Proposed Strategies

Recommendations to improve accessibility were reported in 18 [9,10,13,15,26-28,30,31,34-38,41,43-45] of the 23 included studies [9,10,13,15,26-44]. For example, design-level recommendations were described in 14 studies and included the adoption of universal design principles, collaborative or participatory development processes, and routine accessibility audits during application development.

Several studies recommended improvements to screen reader compatibility, clearer labeling of interface elements, improved navigation flow, and enhanced nonvisual feedback mechanisms. Alternative authentication strategies designed to reduce reliance on visual input were proposed in 5 studies, including tactile- or haptic-based authentication approaches.

User-centered testing and iterative design processes were recommended in 9 studies, particularly to support accessibility maintenance following application updates. Policy- or organizational-level recommendations were reported in 7 studies, including calls for stronger enforcement of accessibility standards, training for financial service providers, and improved regulatory oversight and enforcement for digital financial platforms. Table 3 provides an evidence map summarizing the distribution of reported accessibility barriers across platform types.

**Table 3.** Evidence map of accessibility barriers by platform type and study frequency. The matrix shows the number of included studies reporting each accessibility barrier across different platform types (iOS, Android, web, multiple platforms, and not specified). Cell values indicate study counts. Common barriers include authentication issues (Completely Automated Public Turing test to tell Computers and Humans Apart and one-time password [n=18]), assistive technology compatibility (n=17), and navigation issues (n=15). Multiple studies may contribute to multiple cells because some examine multiple platforms or barrier types. Counts therefore represent the number of studies contributing to each cell rather than mutually exclusive totals.

Barrier description	iOS	Android	Web	Multiple platforms	Not specified	Total
Authentication barriers (CAPTCHA^a^ and OTP^b^)	4	6	5	3	0	18
Assistive technology compatibility issues	5	6	4	2	0	17
Navigation issues	4	7	3	1	0	15
Complex interface layouts	2	5	3	2	0	12
Compatibility issues following updates	2	3	1	1	0	7
Text scaling and readability issues	3	3	1	0	0	7
Insufficient audio feedback	2	3	1	0	0	6
Social or contextual barriers	0	1	0	2	1	4
Physical interface barriers	0	0	0	3	1	4
Technical failures	2	2	0	0	0	4

a CAPTCHA: Completely Automated Public Turing test to tell Computers and Humans Apart.

b OTP: one-time password.

## Discussion

### Principal Findings

This scoping review mapped the published evidence on the accessibility of digital financial apps for people with VI. The evidence reveals persistent, cross-platform accessibility barriers in authentication workflows, screen reader compatibility, and navigation design, alongside facilitators such as consistent assistive technology support, structured navigation, and nonvisual feedback mechanisms that are directly relevant to digital health equity. However, the evidence base remains small, geographically concentrated, and dominated by cross-sectional and qualitative designs. The accessibility barrier-enabler-gap matrix (Table 2) illustrates how these challenges manifest across 5 stages of the user journey, from authentication through ongoing use, and reveals that evidence gaps are most pronounced at the confirmation and ongoing-use stages, where almost no empirical evaluation exists. No study evaluated the effectiveness of an accessibility intervention over time, and the technical evaluation literature has developed largely in isolation from end user experience research. These findings directly address the review objectives by mapping study characteristics, identifying barriers and facilitators, and highlighting evidence gaps.

Authentication represents the most pervasive barrier domain. Visual CAPTCHA, OTP workflows requiring app switching, session timeouts, and inaccessible biometric verification were described across the majority of included studies and across all platform types. This finding is consistent with Schmeelk and Petrie [46], who surveyed visually disabled users in the United Kingdom and the United States and found that password management, multifactor authentication, and biometric systems each present distinct accessibility barriers. Authentication barriers did not operate in isolation. In combination with screen reader incompatibility and complex navigation, these barriers produced cascading failures that prevented independent transaction completion, a prerequisite for secure and autonomous participation in digitally mediated health-relevant services. Multiple studies documented how this forced reliance on sighted intermediaries for tasks as fundamental as logging in, verifying transactions, and making payments [35,36]. Drawing on the concept of “moneywork,” Kameswaran et al [36] characterized this additional, invisible labor required for people with VI to participate in financial systems that sighted users navigate with minimal friction. Griffith et al [47] provided empirical quantification of these costs, demonstrating the time disparity experienced by blind users encountering web accessibility barriers, findings subsequently referenced by the US Department of Justice in its 2024 Americans with Disabilities Act (ADA) Title II rule on web accessibility. The privacy implications are substantial: sharing account details or transaction amounts with intermediaries undermines the financial autonomy digital banking is intended to support.

As outlined in the Introduction, access to financial services via digital technology constitutes a digital determinant of health [6]. Richardson et al [7] proposed a comprehensive framework for digital health equity, identifying digital tool access as a key determinant across multiple ecological levels. The barriers identified in this review, particularly those affecting authentication and independent transaction management, have implications that extend beyond financial autonomy to downstream health-relevant outcomes. When people with VI cannot independently manage digital payments, they face reduced access to health-related financial functions, including insurance management, medication payments, and health savings. Paik et al [48] demonstrated that underrepresentation in digital health data creates feedback loops worsening health disparities, a mechanism that may also operate through financial data exclusion. Several included studies documented participants forgoing digital transactions or reverting to cash specifically because of accessibility barriers [35,42], suggesting that digital financial inaccessibility may function as an upstream determinant of both financial and health inequity. Ha et al [49], in JMIR, demonstrated the feasibility of user-centered design for disability-inclusive digital health platforms, establishing a model that could be extended to financial technology. Integrating financial application accessibility into digital health equity frameworks would acknowledge the role of financial autonomy in health outcomes and align with the World Health Organization (WHO) global strategy on digital health 2020-2025.

Facilitators ranged from platform-level assistive technology support to design-level innovations. Among the most concrete contributions, Alnfiai and Sampalli [10] proposed BraillePassword, a gesture-based authentication method leveraging Braille literacy that directly addresses password-entry barriers. A related innovation, TapCAPTCHA [50], demonstrated that gesture-based CAPTCHA alternatives using audio clips achieved substantially better success rates for users with VIs compared to conventional audio CAPTCHA, offering a concrete solution to the visual CAPTCHA barrier identified repeatedly across included studies. Several studies demonstrated viable offline currency recognition apps integrating text-to-speech output, addressing persistent cash-handling barriers in contexts where tactile denomination features are unreliable. At the system level, Alshayban and Malek [15] demonstrated automated accessibility evaluation at scale, suggesting a pathway toward continuous monitoring rather than reliance on postrelease user complaints. These innovations sit within a growing ecosystem of mobile apps functioning as assistive technologies for people with VI [51], though translation into mainstream financial services remains limited. User-generated workarounds, such as memorizing button locations, preorganizing currency notes by denomination, and switching between multiple apps, also emerged as an important theme, offering practical insight into unmet design needs [35,36].

The barriers identified in this review are consistent with broader digital accessibility research, but their interaction with financial transaction workflows creates a distinctive pattern of risk. In general app accessibility, an unlabeled button may cause frustration; in banking apps, an inaccessible authentication screen can prevent account access entirely, creating financial and privacy vulnerability. Puli et al [12] reviewed financial inclusion for people with disabilities broadly and identified digital inaccessibility as a barrier; this review extends this by mapping the specific mechanisms through which inaccessibility operates for people with VI in financial apps. Hamideh Kerdar et al [21] reviewed digital technology accessibility for people with VI across 49 studies but did not focus on financial apps, confirming the gap this review addresses. In the European context, Borowska-Beszta et al [52] framed digital payment services as a form of assistive technology that can empower consumers with disabilities, a perspective that reframes financial app accessibility not merely as a compliance issue but as an enabler of autonomy. Wilson et al [53], in a systematic review of strategies to advance digital health equity, found that people with disabilities face compounding barriers across multiple equity dimensions, consistent with our finding that authentication, navigation, and labeling barriers compound rather than occur in isolation. Notably, the 2 research streams identified in this review, end user experience studies and technical evaluations, appear largely disconnected, echoing Ortiz-Escobar et al [11] who found limited implementation of user-centered design standards in assistive technology development.

The geographic concentration of evidence in India warrants examination. India’s prominence likely reflects the convergence of a large population with VI, rapid digitalization of financial services through platforms such as Unified Payments Interface, and the postdemonetization context in which the withdrawal of high-denomination notes in 2016 made digital payment adoption nearly mandatory [9,35]. The relative absence of studies from Europe and North America is notable given established regulatory frameworks such as the European Accessibility Act (compliance deadline 2025) and the ADA. Recent evidence from other contexts, however, suggests that the barriers identified in this review are global rather than India-specific: Fayyad and Al-Sinnawi [23] documented persistent financial exclusion of people with VI in Palestine despite national policy commitments; Huang et al [54] identified insufficient legal safeguards for digital financial accessibility in China; Goundar and Sathye [55] found marketplace barriers to financial service access for people with VI in Fiji; and Muuo et al [56] reported that while persons with disabilities in Kenya embrace mobile money, financial institutions lack the knowledge to adapt their products. These findings suggest that regulatory coverage alone does not guarantee accessible implementation and that comparative studies across regulatory environments could help identify which policy mechanisms translate into measurable improvements.

The evidence gaps identified suggest several priorities. First, no included study evaluated the effectiveness of an accessibility intervention over time; longitudinal or pre-post studies assessing the impact of specific design changes on transaction success rates would provide intervention-level evidence. Second, integrating the disconnected technical and end user research streams is essential, as Ara et al [57] found that automated accessibility evaluation alone is insufficient and must be complemented by testing with assistive technology users. Third, participatory design involving users with VIs, guided by emerging best practices [14], should be embedded in financial application development rather than limited to post hoc usability testing. Fourth, the intersectionality of VI with age, digital literacy, and gender remains underexplored; most qualitative studies recruited predominantly male participants. Fifth, low vision was included in fewer studies than blindness, despite representing the majority of the global VI population. Finally, with the emergence of artificial intelligence–powered accessibility solutions [58], future research should examine whether conversational agents, intelligent interfaces, and automated remediation tools can meaningfully reduce the barriers identified in this review.

### Limitations

This scoping review has several limitations. The included studies were predominantly qualitative and conducted in diverse settings, limiting direct comparability. The geographic concentration of many studies in India suggests potential context-specific findings, potentially limiting global generalizability. Additionally, sample sizes in the included studies were often small, and limited quantitative data were available, restricting robust evaluation of the effectiveness of accessibility features. Finally, publication bias toward positive outcomes and the exclusion of gray literature may have influenced the completeness of the evidence. The initial search strategy used a more restrictive 4-block structure. Following peer review, this was revised to 3 blocks (consistent with the Cochrane Handbook Section 4.4.2) and rerun across all databases on February 27, 2026, yielding 23 studies compared to 12 in the original analysis. While the revision strengthened the search, relevant studies indexed under nonstandard terminology or in databases not included in the search may not have been captured. The predominance of qualitative and cross-sectional designs among the included studies also limits the ability to draw causal inferences about the effectiveness of specific accessibility features or interventions.

### Conclusions

This scoping review provides the first focused synthesis of accessibility evidence at the intersection of digital financial services, VI, and digital health equity. Unlike prior reviews examining either ICT accessibility for users with VIs broadly [21] or financial inclusion for disability generally [12], this review identifies the specific mechanisms through which inaccessible financial apps undermine independence, privacy, and financial autonomy. By mapping 23 studies across 12 countries, the review identifies authentication, interface labeling, and navigation as persistent cross-platform barriers; reveals a disconnect between technical design literature and end user experience research; and highlights the absence of longitudinal or intervention-based evidence. The findings carry direct implications for multiple stakeholders. Financial technology developers should prioritize accessible authentication workflows, and evidence that alternatives such as gesture-based CAPTCHAs outperform standard audio CAPTCHAs [50] demonstrates that viable solutions exist. Accessibility auditors should evaluate the full transaction lifecycle, not individual screens. Policymakers implementing the European Accessibility Act, ADA, and comparable frameworks should consider mandatory accessibility certification for financial apps, noting that emerging evidence from China [54], Palestine [23], and Kenya [56] demonstrates that legislative intent does not guarantee accessible implementation. More broadly, integrating financial application accessibility into digital health equity frameworks [6,7] would acknowledge the role of financial autonomy in health outcomes and position this work within the broader digital determinants of health agenda.

## Supplementary material

10.2196/82315 Multimedia Appendix 1 Search strategy including full search strings for each database.

10.2196/82315 Checklist 1 PRISMA-ScR checklist.

## Abbreviations

ADA: Americans with Disabilities Act
ICT: information and communication technology
MeSH: Medical Subject Headings
OTP: one-time password
PRISMA-ScR: Preferred Reporting Items for Systematic Reviews and Meta-Analyses extension for Scoping Reviews
VI: visual impairment
WHO: World Health Organization
