# Supplementary material for: Accessibility of Digital Financial Applications for People With Visual Impairment: Scoping Review
Source: J Med Internet Res. 2026 Jun 23;28:e82315. doi: 10.2196/82315 (PMC13290165; doi:10.2196/82315)
Supplement: Multimedia Appendix 1 [file jmir-v28-e82315-s001.docx]

**Appendix 1: Search strategy including full search strings for each database.**

The initial search was conducted in June 2024 and rerun in February 2026. Searches were limited to English-language publications published between 1995 and 2026. Full search strategies for each database are provided below.

**Note**: Search strategies were adapted for each database to reflect differences in indexing systems, controlled vocabulary, and search syntax.

| **PubMed (MEDLINE)** |
| --- |
| (  "Visually Impaired Persons"[MeSH] OR  "Blindness"[MeSH] OR  "Vision Disorders"[MeSH] OR  "Eye Diseases"[MeSH] OR  "Retinal Diseases"[MeSH] OR  blind*[tiab] OR  "visual* impair*"[tiab] OR  "vision loss"[tiab] OR  "low vision"[tiab] OR  "sight loss"[tiab] OR  "partial sight"[tiab]  )    AND    (  "User-Computer Interface"[MeSH] OR  "Software Design"[MeSH] OR  "Human Engineering"[MeSH] OR  "Assistive Technology"[MeSH] OR  accessib*[tiab] OR  usabil*[tiab] OR  "user experience"[tiab] OR  "user satisfaction"[tiab] OR  interface[tiab] OR  "user interface"[tiab] OR  "interaction design"[tiab] OR  "inclusive design"[tiab] OR  "universal design"[tiab] OR  WCAG[tiab] OR  "Web Content Accessibility Guidelines"[tiab] OR  "screen reader*"[tiab] OR  "assistive technolog*"[tiab] OR  barrier*[tiab] OR  facilit*[tiab] OR  obstacle*[tiab] OR  authentication[tiab] OR  "two-factor authentication"[tiab] OR  "multi-factor authentication"[tiab] OR  MFA[tiab] OR  CAPTCHA[tiab] OR  "one-time password"[tiab] OR  OTP[tiab] OR  biometr*[tiab] OR  "facial recognition"[tiab] OR  "fingerprint recognition"[tiab]  )    AND    (  "Mobile Applications"[MeSH] OR  "Banks"[MeSH] OR  "Electronic Commerce"[MeSH] OR  "Financial Management"[MeSH] OR  "Internet"[MeSH] OR  "Information Technology"[MeSH] OR  "mobile bank*"[tiab] OR  "online bank*"[tiab] OR  "internet bank*"[tiab] OR  "banking app*"[tiab] OR  "bank app*"[tiab] OR  fintech[tiab] OR  "financial technolog*"[tiab] OR  "digital payment*"[tiab] OR  "mobile payment*"[tiab] OR  contactless[tiab] OR  NFC[tiab] OR  "QR payment*"[tiab] OR  "digital wallet*"[tiab] OR  ewallet*[tiab] OR  "mobile money"[tiab] OR  neobank*[tiab] OR  "challenger bank*"[tiab] OR  "digital financial service*"[tiab] OR  DFS[tiab] OR  UPI[tiab] OR  smartphone*[tiab] OR  "mobile app*"[tiab] OR  "mobile technolog*"[tiab] |
| **Scopus** |
| TITLE-ABS-KEY(  blind* OR  "visual* impair*" OR  "vision loss" OR  "low vision" OR  "sight loss" OR  "partial sight"  )  AND  TITLE-ABS-KEY(  accessib* OR  usabil* OR  "user experience" OR  "user satisfaction" OR  interface OR  "user interface" OR  "interaction design" OR  "inclusive design" OR  "universal design" OR  WCAG OR  "Web Content Accessibility Guidelines" OR  "screen reader*" OR  "assistive technolog*" OR  barrier* OR  facilit* OR  obstacle* OR  authentication OR  "two-factor authentication" OR  "multi-factor authentication" OR  MFA OR  CAPTCHA OR  "one-time password" OR  OTP OR  biometr* OR  "facial recognition" OR  "fingerprint recognition"  )  AND  TITLE-ABS-KEY(  "mobile bank*" OR  "online bank*" OR  "internet bank*" OR  "banking app*" OR  "bank app*" OR  fintech OR  "financial technolog*" OR  "digital payment*" OR  "mobile payment*" OR  contactless OR  NFC OR  "QR payment*" OR  "digital wallet*" OR  ewallet* OR  "mobile money" OR  neobank* OR  "challenger bank*" OR  "digital financial service*" OR  DFS OR  UPI OR  smartphone* OR  "mobile app*" OR  "mobile technolog*"  ) |
| **CINAHL** |
| (  (MH "Visual Impairment+") OR  (MH "Blindness+") OR  (MH "Vision Disorders+") OR  (MH "Eye Diseases+") OR  (MH "Retinal Disorders+") OR  TI (blind* OR "visual* impair*" OR "vision loss" OR "low vision" OR "sight loss" OR "partial sight") OR  AB (blind* OR "visual* impair*" OR "vision loss" OR "low vision" OR "sight loss" OR "partial sight")  )  AND  (  (MH "Human Computer Interaction") OR  (MH "Software Design") OR  (MH "Assistive Technology") OR  TI (  accessib* OR  usabil* OR  "user experience" OR  "user satisfaction" OR  interface OR  "user interface" OR  "interaction design" OR  "inclusive design" OR  "universal design" OR  WCAG OR  "Web Content Accessibility Guidelines" OR  "screen reader*" OR  "assistive technolog*" OR  barrier* OR  facilit* OR  obstacle* OR  authentication OR  "two-factor authentication" OR  "multi-factor authentication" OR  MFA OR  CAPTCHA OR  "one-time password" OR  OTP OR  biometr* OR  "facial recognition" OR  "fingerprint recognition"  )  OR  AB (  accessib* OR  usabil* OR  "user experience" OR  "user satisfaction" OR  interface OR  "user interface" OR  "interaction design" OR  "inclusive design" OR  "universal design" OR  WCAG OR  "Web Content Accessibility Guidelines" OR  "screen reader*" OR  "assistive technolog*" OR  barrier* OR  facilit* OR  obstacle* OR  authentication OR  "two-factor authentication" OR  "multi-factor authentication" OR  MFA OR  CAPTCHA OR  "one-time password" OR  OTP OR  biometr* OR  "facial recognition" OR  "fingerprint recognition"  )  )  AND  (  (MH "Mobile Applications") OR  (MH "Banks and Banking") OR  (MH "Electronic Commerce") OR  (MH "Financial Management") OR  TI (  "mobile bank*" OR  "online bank*" OR  "internet bank*" OR  "banking app*" OR  "bank app*" OR  fintech OR  "financial technolog*" OR  "digital payment*" OR  "mobile payment*" OR  contactless OR  NFC OR  "QR payment*" OR  "digital wallet*" OR  ewallet* OR  "mobile money" OR  neobank* OR  "challenger bank*" OR  "digital financial service*" OR  DFS OR  UPI OR  smartphone* OR  "mobile app*" OR  "mobile technolog*"  )  OR  AB (  "mobile bank*" OR  "online bank*" OR  "internet bank*" OR  "banking app*" OR  "bank app*" OR  fintech OR  "financial technolog*" OR  "digital payment*" OR  "mobile payment*" OR  contactless OR  NFC OR  "QR payment*" OR  "digital wallet*" OR  ewallet* OR  "mobile money" OR  neobank* OR  "challenger bank*" OR  "digital financial service*" OR  DFS OR  UPI OR  smartphone* OR  "mobile app*" OR  "mobile technolog*"  )  ) |
| **Web of Science** |
| TS=(  blind* OR  "visual* impair*" OR  "vision loss" OR  "low vision" OR  "sight loss" OR  "partial sight"  )  AND  TS=(  accessib* OR  usabil* OR  "user experience" OR  "user satisfaction" OR  interface OR  "user interface" OR  "interaction design" OR  "inclusive design" OR  "universal design" OR  WCAG OR  "Web Content Accessibility Guidelines" OR  "screen reader*" OR  "assistive technolog*" OR  barrier* OR  facilit* OR  obstacle* OR  authentication OR  "two-factor authentication" OR  "multi-factor authentication" OR  MFA OR  CAPTCHA OR  "one-time password" OR  OTP OR  biometr* OR  "facial recognition" OR  "fingerprint recognition"  )  AND  TS=(  "mobile bank*" OR  "online bank*" OR  "internet bank*" OR  "banking app*" OR  "bank app*" OR  fintech OR  "financial technolog*" OR  "digital payment*" OR  "mobile payment*" OR  contactless OR  NFC OR  "QR payment*" OR  "digital wallet*" OR  ewallet* OR  "mobile money" OR  neobank* OR  "challenger bank*" OR  "digital financial service*" OR  DFS OR  UPI OR  smartphone* OR  "mobile app*" OR  "mobile technolog*"  ) |
| **Business Source Complete** |
| (  TI (blind* OR "visual* impair*" OR "vision loss" OR "low vision" OR "sight loss" OR "partial sight")  OR  AB (blind* OR "visual* impair*" OR "vision loss" OR "low vision" OR "sight loss" OR "partial sight")  )  AND  (  TI (  accessib* OR  usabil* OR  "user experience" OR  "user satisfaction" OR  interface OR  "user interface" OR  "interaction design" OR  "inclusive design" OR  "universal design" OR  WCAG OR  "Web Content Accessibility Guidelines" OR  "screen reader*" OR  "assistive technolog*" OR  barrier* OR  facilit* OR  obstacle* OR  authentication OR  "two-factor authentication" OR  "multi-factor authentication" OR  MFA OR  CAPTCHA OR  "one-time password" OR  OTP OR  biometr* OR  "facial recognition" OR  "fingerprint recognition"  )  OR  AB (  accessib* OR  usabil* OR  "user experience" OR  "user satisfaction" OR  interface OR  "user interface" OR  "interaction design" OR  "inclusive design" OR  "universal design" OR  WCAG OR  "Web Content Accessibility Guidelines" OR  "screen reader*" OR  "assistive technolog*" OR  barrier* OR  facilit* OR  obstacle* OR  authentication OR  "two-factor authentication" OR  "multi-factor authentication" OR  MFA OR  CAPTCHA OR  "one-time password" OR  OTP OR  biometr* OR  "facial recognition" OR  "fingerprint recognition"  )  )  AND  (  TI (  "mobile bank*" OR  "online bank*" OR  "internet bank*" OR  "banking app*" OR  "bank app*" OR  fintech OR  "financial technolog*" OR  "digital payment*" OR  "mobile payment*" OR  contactless OR  NFC OR  "QR payment*" OR  "digital wallet*" OR  ewallet* OR  "mobile money" OR  neobank* OR  "challenger bank*" OR  "digital financial service*" OR  DFS OR  UPI OR  smartphone* OR  "mobile app*" OR  "mobile technolog*"  )  OR  AB (  "mobile bank*" OR  "online bank*" OR  "internet bank*" OR  "banking app*" OR  "bank app*" OR  fintech OR  "financial technolog*" OR  "digital payment*" OR  "mobile payment*" OR  contactless OR  NFC OR  "QR payment*" OR  "digital wallet*" OR  ewallet* OR  "mobile money" OR  neobank* OR  "challenger bank*" OR  "digital financial service*" OR  DFS OR  UPI OR  smartphone* OR  "mobile app*" OR  "mobile technolog*"  )  ) |
| **ProQuest** |
| TI,AB(  blind* OR  "visual* impair*" OR  "vision loss" OR  "low vision" OR  "sight loss" OR  "partial sight"  )  AND  TI,AB(  accessib* OR  usabil* OR  "user experience" OR  "user satisfaction" OR  interface OR  "user interface" OR  "interaction design" OR  "inclusive design" OR  "universal design" OR  WCAG OR  "Web Content Accessibility Guidelines" OR  "screen reader*" OR  "assistive technolog*" OR  barrier* OR  facilit* OR  obstacle* OR  authentication OR  "two-factor authentication" OR  "multi-factor authentication" OR  MFA OR  CAPTCHA OR  "one-time password" OR  OTP OR  biometr* OR  "facial recognition" OR  "fingerprint recognition"  )  AND  TI,AB(  "mobile bank*" OR  "online bank*" OR  "internet bank*" OR  "banking app*" OR  "bank app*" OR  fintech OR  "financial technolog*" OR  "digital payment*" OR  "mobile payment*" OR  contactless OR  NFC OR  "QR payment*" OR  "digital wallet*" OR  ewallet* OR  "mobile money" OR  neobank* OR  "challenger bank*" OR  "digital financial service*" OR  DFS OR  UPI OR  smartphone* OR  "mobile app*" OR  "mobile technolog*"  ) |
| **IEEE Xplore** |
| (  ("Abstract":blind* OR "Abstract":"visual* impair*" OR "Abstract":"vision loss" OR "Abstract":"low vision" OR "Abstract":"sight loss" OR "Abstract":"partial sight")  OR  ("Document Title":blind* OR "Document Title":"visual* impair*" OR "Document Title":"vision loss" OR "Document Title":"low vision")  OR  ("Author Keywords":blind* OR "Author Keywords":"visual impairment" OR "Author Keywords":"low vision")  )  AND  (  ("Abstract":accessib* OR "Abstract":usabil* OR "Abstract":"user experience" OR "Abstract":"user satisfaction" OR "Abstract":interface OR "Abstract":"user interface" OR "Abstract":"interaction design" OR "Abstract":"inclusive design" OR "Abstract":"universal design" OR "Abstract":WCAG OR "Abstract":"screen reader*" OR "Abstract":"assistive technolog*" OR "Abstract":barrier* OR "Abstract":authentication OR "Abstract":"two-factor authentication" OR "Abstract":"multi-factor authentication" OR "Abstract":CAPTCHA OR "Abstract":"one-time password" OR "Abstract":OTP OR "Abstract":biometr*)  OR  ("Author Keywords":accessib* OR "Author Keywords":usabil* OR "Author Keywords":"screen reader*" OR "Author Keywords":authentication)  )  AND  (  ("Abstract":"mobile bank*" OR "Abstract":"online bank*" OR "Abstract":"internet bank*" OR "Abstract":"banking app*" OR "Abstract":"bank app*" OR "Abstract":fintech OR "Abstract":"financial technolog*" OR "Abstract":"digital payment*" OR "Abstract":"mobile payment*" OR "Abstract":"digital wallet*" OR "Abstract":"mobile money" OR "Abstract":"financial service*" OR "Abstract":UPI OR "Abstract":"mobile app*" OR "Abstract":smartphone*)  OR  ("Author Keywords":"mobile banking" OR "Author Keywords":"digital payment*" OR "Author Keywords":"financial technology")  ) |
